# Predicting Systemic Health Features from Retinal Fundus Images Using Transfer-Learning-Based Artificial Intelligence Models

**DOI:** 10.3390/diagnostics12071714

**Published:** 2022-07-14

**Authors:** Nergis C. Khan, Chandrashan Perera, Eliot R. Dow, Karen M. Chen, Vinit B. Mahajan, Prithvi Mruthyunjaya, Diana V. Do, Theodore Leng, David Myung

**Affiliations:** 1 Byers Eye Institute at Stanford, Department of Ophthalmology, Stanford University School of Medicine, Palo Alto, CA 94305, USA; nckhan@stanford.edu (N.C.K.); cperera@stanford.edu (C.P.); eliotdow@stanford.edu (E.R.D.); karenc5@stanford.edu (K.M.C.); vinit.mahajan@stanford.edu (V.B.M.); prithvi9@stanford.edu (P.M.); dianado@stanford.edu (D.V.D.); tedleng@stanford.edu (T.L.); 2 Department of Ophthalmology, Fremantle Hospital, Perth, WA 6004, Australia; 3 VA Palo Alto Health Care System, Palo Alto, CA 94304, USA

**Keywords:** diabetic retinopathy, artificial intelligence, transfer learning, retinal imaging

## Abstract

While color fundus photos are used in routine clinical practice to diagnose ophthalmic conditions, evidence suggests that ocular imaging contains valuable information regarding the systemic health features of patients. These features can be identified through computer vision techniques including deep learning (DL) artificial intelligence (AI) models. We aim to construct a DL model that can predict systemic features from fundus images and to determine the optimal method of model construction for this task. Data were collected from a cohort of patients undergoing diabetic retinopathy screening between March 2020 and March 2021. Two models were created for each of 12 systemic health features based on the DenseNet201 architecture: one utilizing transfer learning with images from ImageNet and another from 35,126 fundus images. Here, 1277 fundus images were used to train the AI models. Area under the receiver operating characteristics curve (AUROC) scores were used to compare the model performance. Models utilizing the ImageNet transfer learning data were superior to those using retinal images for transfer learning (mean AUROC 0.78 vs. 0.65, *p*-value < 0.001). Models using ImageNet pretraining were able to predict systemic features including ethnicity (AUROC 0.93), age > 70 (AUROC 0.90), gender (AUROC 0.85), ACE inhibitor (AUROC 0.82), and ARB medication use (AUROC 0.78). We conclude that fundus images contain valuable information about the systemic characteristics of a patient. To optimize DL model performance, we recommend that even domain specific models consider using transfer learning from more generalized image sets to improve accuracy.

## 1. Introduction

Centuries before the era of modern medicine and biotechnology, the eyes were philosophically and spiritually distinguished from among all other organs as being “windows to the soul”. Today, the eyes are biologically understood to be the only human structure with an internal anatomy, vasculature, and neural tissue structure that can be directly and non-invasively observed from the outside [1]. Ocular imaging modalities that take advantage of this, such as fundoscopy and optical coherence tomography (OCT), have become standard tools for ophthalmologic clinical practice, disease diagnosis, and management [2,3,4]. The utility of ocular imaging, in particular retinal imaging, is now expanding further as artificial intelligence (AI) drives the discovery of new ocular manifestations of systemic health and disease.

Using standalone, high resolution digital fundus and OCT photographs, artificial intelligence models have demonstrated the ability to diagnose a variety of retinal and ophthalmic diseases [5], including diabetic retinopathy [6,7,8,9,10,11], retinopathy of prematurity [12,13], age-related macular degeneration features [14,15,16,17,18], glaucoma [19], and macular telangiectasia [20]. Features of retinal disease such as retinal detachment and retinal vein occlusion are identifiable as well [21,22]. Clinical use of AI-based tools for diabetic retinopathy detection has recently commenced after FDA clearance of IDxDR (Digital Diagnostics; Coralville, IA, USA) in 2018 [23,24,25].

The retina is increasingly being recognized as a medical “window” that extends beyond ocular disease. To this end, the potential of AI models to capture and uncover biomarkers of systemic health and disease, rather than simply ophthalmologic health and disease, from retinal imaging is being explored [26]. Alterations in key retinal features have already been associated with numerous prevalent disease processes [27]. Retinal microvascular changes have been linked to coronary heart disease, hypertension, kidney disease, and stroke [28,29,30,31,32,33,34]. In addition, as the retina itself is an extension of the central nervous system, retinal nerve fiber layer thickness and retinal vessel morphology changes have been found to be predictive of dementia and neurodegenerative illnesses like Parkinson’s and Alzheimer’s disease [35,36,37,38,39,40]. Fundus images can even be predictive of asymptomatic white matter hyperintensities [41]. Newly developed deep learning (DL) models are capable of predicting cardiovascular health parameters such as systolic blood pressure, diastolic blood pressure, BMI, hemoglobin A1c (HbAlc), and current smoking status from fundus imaging alone [42]. Anemia has also been reportedly detected by DL models [43]. A diagnosis of Alzheimer’s disease was predicted by a convolutional neural network (CNN) and a machine learning model based on retinal imaging data alone, and both models performed comparably to a model unblinded to patient electronic medical record (EMR) data [44,45]. Even gender, a general health feature never associated with retinal features before, can now be accurately predicted from retinal fundus photographs using a DL model [46].

DL models using convolutional neural networks are the best performing architectures for image classification tasks, particularly since the advent of ImageNet, a general image database with over 14 million annotated images that fall under 20,000 object categories (i.e., cars, fruits, cats, etc.) [47,48]. Typically, DL models developed for the prediction of systemic health features from retinal images are pre-trained on a dataset of pre-labelled retinal fundus images: training and testing datasets are extracted from the same knowledge distribution. In contrast, transfer learning techniques allow for training and testing datasets to be drawn from different knowledge or content distributions [49]. The uppermost classifier layers of a CNN originally trained on another dataset can be dropped and fine-tuned to classify a new set of target images during the transfer learning process [50]. Recent investigations suggest transfer learning may be particularly advantageous for medical image classification tasks [51,52].

Here, we are seeking to predict novel systemic health features from retinal fundus images. We will also compare the accuracy of two different models of CNN construction: (1) an AI model pre-trained using transfer learning: on general images from the ImageNet database only and (2) an AI model pre-trained on retinal images alone. We hypothesize that a DL model constructed by pre-training with general images will perform best at systemic feature extraction as its early layers are likely to have learned more generalizable features.

## 2. Materials and Methods

### 2.1. Dataset and Design

A total of 1277 de-identified retinal fundus images were obtained from 760 patients previously diagnosed with diabetes mellitus (650 right eyes and 627 left eyes; see Table 1 for further demographic information). Across 790 encounters at the Stanford Healthcare and/or the Stanford University Health Alliance network primary care clinics in the San Francisco Bay Area between March 2020 and March 2021, retinal fundus images were taken as part of patients’ routine diabetic retinopathy screening. Only images from adult patients (>18 years old) were included in this study. This study was approved by the Stanford University Institutional Review Board (no. 57104).

### 2.2. Materials

Retinal images were obtained with the CenterVue DRS fundus camera (Hillrom Inc., Chicago, IL, USA) and the TopCon NW400 fundus camera (Welch Allyn Inc., Skaneateles Falls, NY, USA) at primary care clinics.

### 2.3. Procedures

#### Ground Truth Labeling

**Figure 1:**
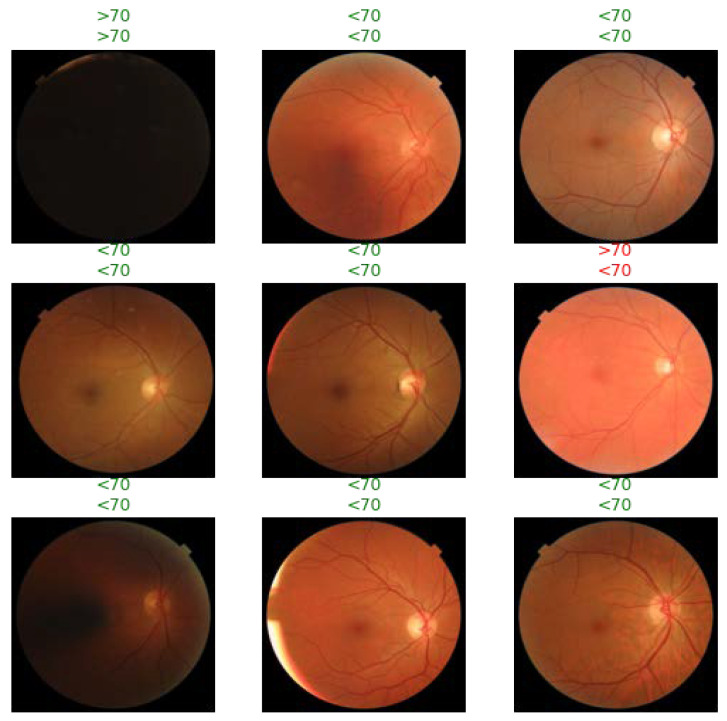
Representative fundus imaging with age > 70 ground-truth labeling and ImageNet-pretrained model classification. Above each fundus image, the first row of data contain the ground truth extracted from patient EMR and the second row contains the AI model pre-trained on ImageNet’s classification. Green and red indicate agreement and disagreement between the AI model and ground truth, respectively. For example, the fundus image in the top row on the far right was correctly predicted to be from a patient under 70 years of age (see the concordance between the ground truth and AI classification), whereas the fundus image in the second row far right was incorrectly predicted by the AI model.

The DL models were trained to predict 12 systemic health features from the retinal image dataset: gender (male or female), ethnicity (Caucasian or non-Caucasian), age (above or below 70 years of age), LDL (above or below 130), HDL (above or below 40), smoking status, cardiac disease (present or absent), HbA1c (above or below 6.5%), hypertension (present or absent), angiotensin receptor blocker (ARB) use, angiotensin-converting enzyme inhibitor (ACEi) use, and aspirin use. All lab values were measured within 1 year of the date of the fundus image and were excluded if not available within the specified time frame. The ground truth of patient lab values, comorbid diagnoses, medication history, and general health information were extracted from the EMR and were used to assign labels to the image set. See Figure 1 for a representative patient fundus image with age > 70 ground truth labeling.

### 2.4. Dataset Subdivision

To develop the DL models, the dataset of 1277 fundus images was randomly split into a training and testing set. Here, 80% of the original dataset (totaling 1021 randomly selected images) were utilized as a training set. The remaining 20% of images in the dataset (256 images) were used as a testing set in the final analysis. This was done in a stratified manner for each of the systemic features explored to ensure missing data would not affect the analysis.

### 2.5. Dataset Preprocessing

De-identified imaging and clinical history data were first linked using an anonymized research ID as per the IRB protocol. Images were then resized to 224-pixel squares as required for the chosen model architecture input. Data augmentation techniques such as cropping, warping, and brightness/contrast adjustments were used during batch preparation for each epoch of model training to encode variance in the images.

### 2.6. Model Training and Testing

**Figure 2:**
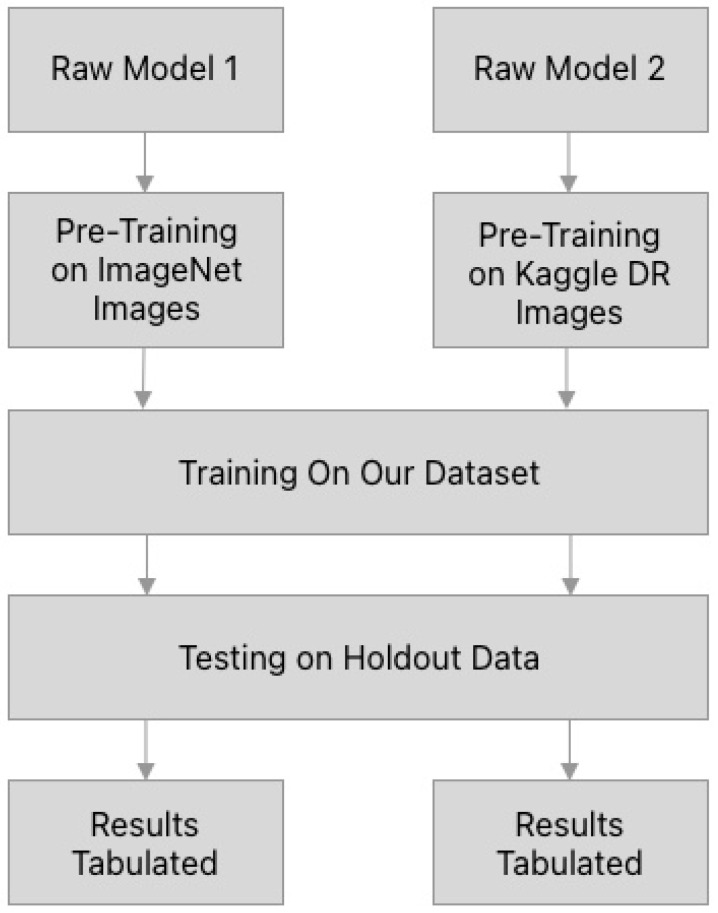
Visual representation of the methodology used to construct both the ImageNet and retinal image pretrained DL models.

The FastAI package was used in a Python environment to develop the AI models. These are based on PyTorch as the underlying framework, with standardized ImageNet pre-training weights used as necessary. See the flowchart in Figure 2 for a visual representation of the model construction.

*Model 1*—This model was created using DenseNet 201 architecture. It was initialized using pre-trained weights publicly available utilizing the ImageNet database. The head layers were then removed to prepare the model as a pre-trained model ready for use in the task at hand.

*Model 2*—This model was created using DenseNet 201 architecture. A total of 35,126 images were acquired from an online dataset of publicly available diabetic retinopathy with associated grades provided by EyePACS [53]. The model was then trained to predict the outcome of diabetic retinopathy images until convergence. The head layers were then removed to prepare the model as a pretrained model ready for use in the task at hand.

Each model was then trained using optimized cyclical learning rates, with the head layer initially optimized, then the deeper layers were also allowed to have their weights adjusted in a weighted fashion, with more superficial layer weights being modified most. A cyclical learning rate was used to maximize learning until convergence and then each model was evaluated on the hold out test dataset.

### 2.7. Statistical Analysis

**Figure 3:**
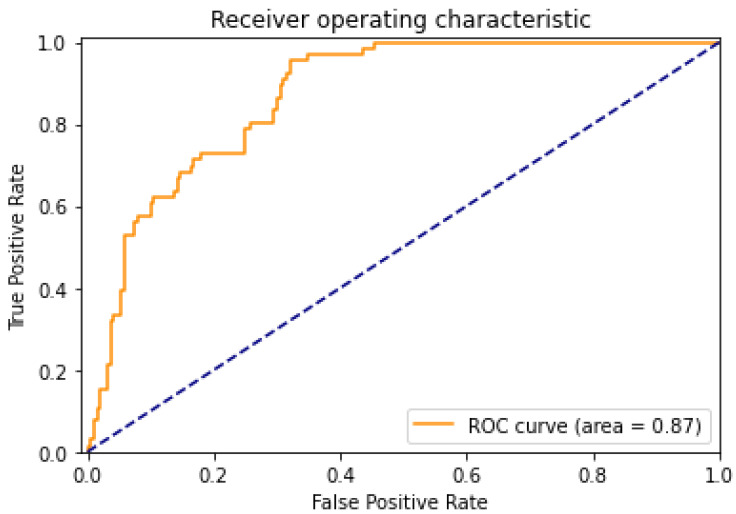
Representative receiver operating characteristic (ROC) curve for ImageNet-pretrained AI model classification of patient Age > 70 (yellow line). Note the ROC curve area, which indicates the achieved area under the ROC (AUROC). The performance of a hypothetical random classifier (AUROC = 0.5) is represented by the blue dashed line.

Statistical measures were computed using Python. Model performance metrics such as the area under the receiver operating characteristic (ROC) curve (AUROC), sensitivity, specificity, and optimized F1 score (see Figure 3 for a representative ROC curve based on age > 70 classification) were calculated. A Student’s *t*-test was used to calculate *p*-values where relevant.

## 3. Results

### 3.1. Dataset Characteristics

Of the 760 total participants, 54.7% were male and 45.3% were female, with a combined median age of 60 and a mean of 59.5 years. Prior to imaging, 88%, 76.8%, 91.6%, and 11.8% of participants had previously been diagnosed with cardiac disease, stroke, hypertension, or diabetic retinopathy, respectively. The largest age group in this dataset was the 60–69 years of age group, which consisted of 25.7% of the total patients. More patients (31.5%) identified as African American than with any other racial group in this dataset and 69.2% described themselves as non-Hispanic (see Table 1).

### 3.2. AI Models Can Predict Systemic Health Features from Fundus Imaging Alone

**Figure 4:**
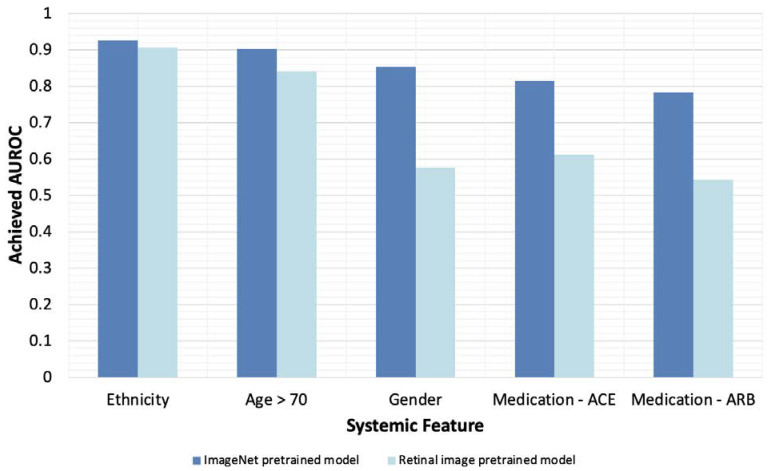
Five systemic features for which the ImageNet pretrained AI model achieved the highest classification accuracy based on AUROC. Dark blue represents the ImageNet pretrained model; light blue represents the retinal image pretrained model.

Out of the two AI models, the best performing model across all four measured performance metrics was the model pretrained on the ImageNet database. Of the 12 systemic health features of interest, the five features for which the ImageNet-pretrained AI model achieved the highest classification accuracy are plotted in Figure 4. Ethnicity was the systemic health feature corresponding to the model’s highest AUROC (0.926), followed by age (0.902), gender (0.852), ACEi medication use (0.815), and ARB medication use (0.783). The model achieved an AUROC in the range of 0.766 to 0.687 for the remaining seven systemic features (see Table 2). Eight out of the 12 features were predicted with an AUROC above 0.700. The model’s optimized F1 score was the highest for age (0.873) and ethnicity (0.871), but lowest for HbA1c (0.669). Sensitivity and specificity across systemic health features ranged from 0.862 to 0.625 and 0.886 to 0.598, respectively. Sensitivity was highest for the classification of age and lowest for cardiac disease. Specificity was highest for ethnicity and lowest for cardiac disease.

### 3.3. Pretraining with General Images Optimizes Model Performance

**Figure 5:**
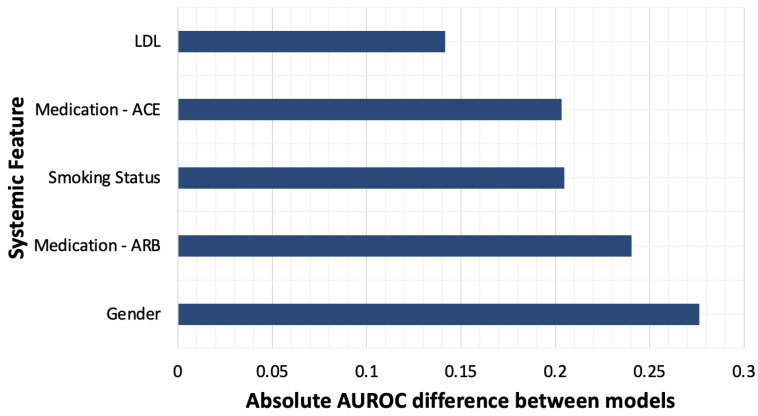
Five systemic health features for which the classification accuracy differed the most between the two AI models based on AUROC.

The AI model pre-trained on ImageNet images performed significantly better than the model pre-trained on retinal images across all 12 systemic feature classifications (*p*-value < 0.001; see Table 3). The mean AUROC obtained across all 12 features for the ImageNet pretrained model and the retinal image pretrained model were 0.78 and 0.65, respectively. Figure 4 plots the ImageNet pretrained model’s AUROC values for the five systemic features that were predicted most accurately, alongside AUROC values achieved by the retinal image pretrained model. The absolute differences in achieved AUROC between the two models differed across systemic features (Figure 5), with the greatest absolute difference in performance observed for gender classification. The magnitude of the absolute AUROC difference between models was least for the ethnicity classification. In addition, the pretrained ImageNet model produced consistently higher sensitivity and specificity values across all systemic feature categories compared with the untrained model.

### 3.4. AI Models Attend to Fundus Images in a Physiologically Valid Manner

**Figure 6:**
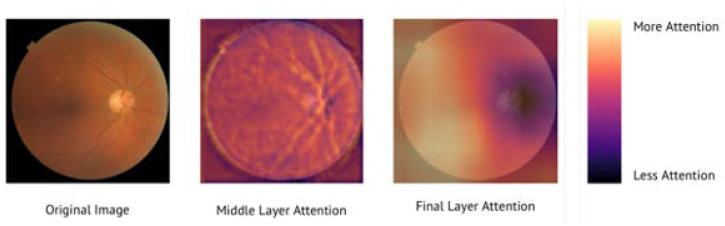
Gradient activation map. This map demonstrates which region of the image the AI model is attending to at various CNN layer depths. The original fundus image for analysis is on the far left. The second image demonstrates which areas of the image the model is paying the greatest attention to in the middle layers of the model. The third image demonstrates the regions of the fundus image that the final layer of the model is paying most attention towards. The scale on the far right indicates the per-pixel degree of model attention from most attention to least attention.

Gradient activation maps corresponding to the middle layer and the final, deepest layer of the ImageNet-pretrained AI model are shown in Figure 6. The middle layer of the AI model pays particular attention to the retinal vessel structure, tortuosity, and caliber. By the final layer, the AI model evolves to primarily attend to features present in the general macular and inferior arcade area of the fundus, while paying less attention to the optic disc region.

### 3.5. Feature Categories with Missing Data

For seven feature categories, all 1277 fundus images had associated patient health data available in the EMR. Smoking status, HbA1c, ethnicity, LDL, and HDL data were not comprehensively available for all fundus images: 97.6%, 92.6%, 92.5%, 88.4%, and 22.7% of images had corresponding EMR data, respectively (see Table 4).

## 4. Discussion

This study demonstrates that DL models can reasonably predict a diverse set of clinically relevant features related to patient demographics, medication use, and general systemic health state from retinal fundus images alone. Patient ethnicity, age, gender, ACE inhibitor use, and ARB medication use were classified with particularly high accuracy based on AUROC from the receiver operating characteristic curve. To our knowledge, ethnicity, ACE inhibitor, and ARB medication use have not previously been predicted with an AI model solely from retinal fundus images. Our results also indicate that pre-training DL models on a general image dataset, such as ImageNet, leads to a significantly improved performance compared with DL models pretrained on retinal images across all 12 investigated systemic health features. Furthermore, the AI models demonstrate a physiologically valid method of “viewing” retinal images across layers: paying attention to image features we would expect to be significant such as vessel structure and macular integrity, while notably not attending to incidental camera, lens, or image artifacts.

### 4.1. Clinical Significance

The clinical utility of AI-based prediction of patient demographic features and medication use is naturally limited, but our findings strongly suggest there is more to the retina than initially meets the eye. Taken together with the findings from Poplin et al. and Korot et al., the accurate prediction of features such as ethnicity, gender, and age, which have never previously been connected to specific retinal neurovascular changes, is a promising indication that there is meaningful, predictive information contained in the retina that has yet to be discovered and understood [39,43]. Furthermore, given that features with direct effects on systemic disease processes, such as LDL and HbA1c, were capable of being extracted suggests that novel disease biomarkers have the potential to eventually be identified with the help of DL models. Using AI as a tool for biomarker discovery within the retina will both improve our understanding of the pathogenesis of highly prevalent diseases and will allow for less invasive, low-cost, and more accessible patient screening during ophthalmologic examination, with the eventual goal of earlier and more accessible disease detection across various patient populations. This will ultimately improve holistic patient care beyond ophthalmology by allowing patients to be diagnosed with various conditions in a non-invasive manner, which can be done by a trained technician and automated AI analysis. Future studies should apply similar DL models to the prediction and classification of systemic features relevant to other pathologies that have both a high public health burden and a potential ophthalmic manifestation beyond cardiovascular disease and diabetes, such as Alzheimer’s disease [26].

### 4.2. Advantages of Transfer-Learning Techniques

Notably, the DL model solely trained on retinal fundus images performed significantly more poorly than the transfer-learning model that was trained on the general ImageNet database beforehand. Typically, in AI models, the earlier layers of the models are focused on identifying simple features such as edges, straight lines, and curves. As the layers progress, they then start to identify objects with increasing complexity: from simple shapes such as circles, through to more abstract objects such as faces [54]. The activations from the final layers are then used to make the final decision on what the original image is most likely to be based on the model architecture. A simple way of explaining this is to imagine the model like a small child that is learning to recognize patterns. First, by teaching the child to understand the concept of a variety of different simple shapes and objects, the child learns to recognize basic patterns. Subsequently, the child will be better at recognizing more subtle differences within the same topic (e.g., differentiating specific animals) as they have understood the basics of how to identify various shapes and objects as a first step.

We hypothesize that a model pre-trained on ImageNet data has been exposed to a far greater degree of heterogeneity in the training images it has seen—and, as such, the earlier layers of the model are likely to have a wider discriminatory ability to identify a larger degree of features in an image. The model pre-trained on retinal images has seen more retinal images; however, it has only learned to identify features relevant to diabetic retinopathy in fundus images. When this model is then forced to re-learn new outputs, it is less likely to have the early discriminatory layers, which will allow it to identify new features it has not had previous exposure to. As such, the model, which has pre-trained on a wider variety of images, has an improved performance in the new task. We believe this may help future researchers in choosing the architecture of their model for domain specific tasks where it is tempting to use domain specific images to develop their model with pre-training. In reality, it may be better to pre-train their models on a more diverse, heterogeneous dataset such as the ImageNet dataset.

### 4.3. Addressing Bias in Artificial Intelligence Models

Another strength of this paper and dataset we present is that our patient group contains a diverse group of ethnic and racial origins. In our cohort, 32.0% were African American, 25.6% were Asian, and over 21% identified as Hispanic. The inclusive nature of this dataset is due to the catchment area of the clinics from which the retinal images were sourced. The issue of racial bias in AI has been identified in multiple papers in the literature [55,56] and has the potential to lead to the development of AI models that perform well in the patient populations on which they are trained (usually Caucasian populations) and to underperform in other patient groups, leading to inequitable access to and utility of these technologies in minority populations. We believe the inclusive nature of our dataset may result in an AI model that generalizes better to different ethnic/racial groups.

### 4.4. Limitations and Future Directions

The study results were limited by several factors. First and foremost, data availability. Currently, our models are designed for binary prediction tasks due to the fact that a prediction task with a numerical output on a spectrum of possible values requires a larger and more diverse training and testing dataset. That our DL models could accurately classify typically numerical systemic health features such as age, LDL, and HbA1c suggests that similar DL models will successfully predict similar features utilizing a larger dataset with continuous variables. A future study should be undertaken on such a larger image dataset to test this hypothesis. Additionally, the present study was based on a set of patients who all have diabetes mellitus, as retinal imaging is a routine part of diabetic disease management. However, to expand the potential systemic health features and disease processes of interest, future studies should obtain routine fundus images from a more generalizable and varied set of patients, including patients who do not have diabetes. Given that fundus imaging is non-invasive and low risk for patients, this should be relatively feasible. Furthermore, the sample size overall of patients with corresponding health data available in the EMR was lower than anticipated—various amounts of missing data were observed across each of the 12 systemic feature categories. A more robust dataset would have likely yielded better model performance across those features with particularly high levels of missing data. Finally, in our study, we chose to use each fundus photo from a patient as a separate data point. Firstly, there are heterogeneity in this data, with each eye possibly showing different features. Secondly, due to limitations in sample size, we did not want to further reduce the dataset, which would limit the amount of data available for training/validation. Future studies with larger numbers would be able to test different combinations of using one eye, either eye, or both eyes for analysis.

Future research directions include using general pre-trained DL models to predict changes in patient systemic health features longitudinally rather than exclusively at a single point in time. Such prognostic information would assist clinicians with predictions of a patient’s disease course: patients found to be at a greater risk of a more severe clinical course might be targeted for earlier implementation of medical and lifestyle interventions. Investigating this would require the inclusion of time series data for each systemic feature in the dataset and constructing a longitudinal cohort of patients. In addition, based on our finding, a significant improvement in DL model performance with general image transfer learning, future researchers should consider utilizing general pretrained models even in specialized use-case scenarios.

## 5. Conclusions

By constructing a series of AI models, we were able to demonstrate that fundus images contain valuable information about the systemic condition of a patient, and that these systemic features can be predicted with a reasonable degree of accuracy using a well-constructed model. We were also able to demonstrate that the use of more generalized datasets such as ImageNet for pre-training, as opposed to using retinal images alone for pre-training, results in a model with improved accuracy to predict these systemic features from fundus images.

## Figures and Tables

**Table 1:** Participant demographics. Information pertaining to patient age, sex, race, ethnicity, and comorbidity status are outlined below.

Demographic Feature	N	Proportion of Dataset (%)
Unique participants	760	–
Total fundus images	1277	–
Right eyes	650	50.9
Left eyes	627	49.1
**Sex**		
Male	432	54.7
Female	358	45.3
**Age (years)**		
20–29	23	2.9
30–39	59	7.5
40–49	130	16.5
50–59	196	24.8
60–69	203	25.7
70–79	126	15.9
80–89	46	5.8
90–99	7	0.9
**Race**		
Asian	202	25.6
African American/Black	253	32
White	68	8.6
Native American/Pacific Islander	18	2.3
Other/Unknown	249	31.5
**Ethnicity**		
Hispanic/Latino	173	21.9
Non-Hispanic/Latino	547	69.2
Other/Unknown	70	8.9
**Comorbidities**		
Cardiac Disease	669	88
Stroke	584	76.8
Hypertension	696	91.6
Diabetic Retinopathy	90	11.8

**Table 2:** ImageNet-pretrained AI model performance. Achieved area under the receiver operating characteristic curve (AUROC), optimized F1 score, sensitivity, and specificity are listed. Systemic features are ordered by descending AUROC.

Systemic Feature	AUROC	Optimized F1 Score	Sensitivity	Specificity
Ethnicity	0.926	0.871	0.86	0.886
Age > 70	0.902	0.873	0.862	0.869
Gender	0.852	0.758	0.742	0.774
Medication—ACEi	0.815	0.804	0.811	0.791
Medication—ARB	0.783	0.707	0.7	0.708
LDL	0.766	0.718	0.694	0.714
HDL	0.756	0.711	0.692	0.722
Smoking status	0.732	0.697	0.632	0.713
HbA1c	0.708	0.669	0.638	0.634
Cardiac disease	0.7	0.669	0.625	0.598
Medication—Aspirin	0.696	0.681	0.673	0.685
Hypertension	0.687	0.695	0.643	0.623

**Table 3:** Comparing ImageNet pretrained and retinal image pretrained model performances. The achieved area under the receiver operating characteristic curve (AUROC) for each of the 12 systemic features are listed. The mean AUROC achieved across all features was found to be statistically significant between the two models (*p* < 0.001).

Systemic Feature	AUROC of ImageNet Pre-Trained Model	AUROC of Retinal Image Pre-Trained Model
Gender	0.852	0.576
Medication—ARB	0.783	0.542
Smoking Status	0.732	0.528
Medication—ACEi	0.815	0.612
LDL	0.766	0.624
Hypertension	0.687	0.585
HDL	0.756	0.667
Cardiac Disease	0.7	0.623
HbA1c	0.708	0.64
Age > 70	0.902	0.84
Medication—Aspirin	0.696	0.638
Ethnicity	0.926	0.907
**Mean AUROC**	0.777	0.648

**Table 4:** Fundus images with corresponding electronic medical record (EMR) feature data. For each of the 12 systemic features of interest, the number of fundus images from among the complete set of 1277 with available corresponding information about the EMR is listed.

Systemic Feature	Images with Corresponding Patient Data	Images without Corresponding Patient Data
Ethnicity	1182	95
Gender	1277	0
LDL	1129	148
HDL	291	986
Smoking status	1247	30
Age > 70	1277	0
Cardiac disease	1277	0
HbA1c	1183	60
Hypertension	1277	0
Medication—ARB	1277	0
Medication—ACEi	1277	0
Medication—Aspirin	1277	0

## Data Availability

Data used to train Model 1 are available upon request from the corresponding author due to personal health information restrictions. Data utilized to train Model 2 are publicly available online: https://www.kaggle.com/c/diabetic-retinopathy-detection (accessed on 23 June 2022).
